# *Humulus lupulus* Cone Extract Efficacy in Alginate-Based Edible Coatings on the Quality and Nutraceutical Traits of Fresh-Cut Kiwifruit

**DOI:** 10.3390/antiox10091395

**Published:** 2021-08-30

**Authors:** Katya Carbone, Valentina Macchioni, Greta Petrella, Daniel Oscar Cicero, Laura Micheli

**Affiliations:** 1 CREA-Research Centre for Olive, Fruit and Citrus Crops, Via di Fioranello 52, 00134 Rome, Italy; valentina.macchioni14@gmail.com; 2 Department of Chemical Sciences and Technologies, University of Rome “Tor Vergata”, Via della Ricerca Scientifica, 00133 Rome, Italy; petrella@scienze.uniroma2.it (G.P.); cicero@scienze.uniroma2.it (D.O.C.); laura.micheli@uniroma2.it (L.M.)

**Keywords:** hop, edible coatings, postharvest treatments, fresh-cut fruit, *Actinidia deliciosa*

## Abstract

In this work, an innovative coating strategy that is able to prolong the shelf-life of fresh-cut kiwifruit was proposed, and the effectiveness of the procedure was evaluated for a period of ten days under cold storage (4 °C). Alginate (2% *m*/*v*) functionalized with green extracts from hop (*Humulus lupulus* L.) cones (HE; 0.5 and 1%, *v*/*v*) was used as a coating material in order to assess the best performing strategy, leading to the most stable product. At the concentrations used to formulate the edible coatings, no contribution related to hop bitterness on the final product was recorded. The results were compared to control samples (without edible coating and coated only with alginate at 2% *m*/*v*). The plant extract was characterized by its main chemical traits and by ^1^H NMR profiling, revealing the presence of antioxidant and antimicrobial bioactive compounds (i.e., alpha and beta hop acids, xanthohumol). Furthermore, the characteristics of the samples during cold storage were evaluated by physico-chemical (i.e., weight loss, soluble solid content, titratable acidity, pH, color attributes) and nutraceutical (i.e., total polyphenol, ascorbic acid content, total carotenoids, chlorophylls) traits. The results showed that the incorporation of hop extracts into the edible coatings tested was able to preserve the quality and nutraceutical traits of fresh-cut kiwifruit during cold storage, thus prolonging their shelf life and marketability.

## 1. Introduction

The sector of fruit and vegetables is of absolute importance in Europe, where, with only 3% of the area under cultivation, it produces almost 21% of the value of total agricultural production in the European Union. Leading countries in this sector are Spain and Italy, which produce more than 45% of the European fresh fruit and vegetables [1]. Nevertheless, even though the daily consumption of fresh fruits and vegetables per capita in Europe increased by 4% in 2018, compared to 2017 levels (www.freshfel.org, accessed on 12 June 2021), it is still below the minimum daily consumption of 400 g recommended by the World Health Organization (WHO). This behavior may be justified by the lack of correspondence between the quality desired by the consumer and the one that is actually perceived by the latter, by the price target, and, above all, by the reduced convenience associated with the consumption of fresh fruit, especially in industrialized countries where prepared foods are increasingly used [2]. It is precisely because of this evidence that the fruit and vegetable industry is progressively moving towards fresh-cut or ready-to-eat products. The IV range fruit and vegetable sector in Italy has a turnover of about EUR 750 million, involving about 200 processing companies and more than 2000 farms, with a growth rate of 5.9% in 2018. About 19.4 million consumers buy these products regularly, and they are also willing to spend more in order to have quality service and safer products. In addition, due to the COVID-19 pandemic, consumer interest in those products that support the overall maintenance of health and wellness has increased, driving consumption toward safer and healthier foods. However, a higher service content corresponds to a greater perishability compared to the initial product: the damage caused during cutting operations produces a series of alterations (i.e., oxidative browning, loss of consistency and increased susceptibility to microorganisms, reduced nutritional value) that lead to a reduction in the useful life of the product, and that make it necessary to implement additional technologies aimed at obtaining a shelf-life compatible with commercial distribution [3]. In addition, COVID-19-induced supply chain disruptions have reinvigorated the drive for shelf life-enhancing food packaging (www.freshfel.org, accessed on 12 June 2021). Several strategies are available to maintain the quality of IV range products: low temperatures (0–4 °C), heat (either used dry, between 35 and 40 °C, or in the form of brief immersion in hot water, up to 50–53 °C), and the use of modified atmospheres are among the most widely adopted physical systems (often in synergy), while chemical systems involve the inhibition of enzymes responsible for browning (polyphenol oxidase, PPO) or the removal or replacement of the substrates on which these enzymes act. However, in recent years, international research has been exploring innovative and sustainable technological solutions dedicated to the IV range sector [3]. Among these, an increasing number of scientific studies are focused on the formulation of edible coatings (ECs)—either simple or functionalized with natural compounds with high biological activity—designed to extend both the shelf life of fresh-cut products and their sensory and nutritional profile, which can even be enriched with these coatings, especially for the nutraceutical component [4,5]. ECs are generally obtained from a triphasic mixture consisting of: (i) a high molecular weight biopolymer, necessary to form a continuous structural matrix with a good degree of cohesion; (ii) a plasticizer, which is essential to increase the flexibility and durability of the material; and (iii) a food grade solvent, which acts as a dispersing agent for the macromolecules. To realize the coating, a thin layer of edible material was coated directly on the food surface, applied in liquid form (film-forming solution/dispersion), usually by immersing (dipping) or spraying. Coating properties change depending on the material with which it is formulated. Each class of compounds has specific properties, advantages, and limitations. Generally, polysaccharides, proteins, and lipids are used as biopolymers in the formulation of an EC, used alone or in mixtures with each other [6]. Among these, polysaccharide-based ECs are the most studied [5,7], and alginate is one of the most evaluated for coating [8].

In addition, due to their chemical nature, these ECs have the ability to carry various active substances, such as plant extracts rich in phytochemical compounds, which give them significant antioxidant and antimicrobial activities [9]. Among plant extracts, those from hop have recently gained great attention from the scientific community due to the presence of a plethora of bioactive compounds, such as polyphenols, prenylflavonoids, bioactive pigments, and bitter acids [10]. The cultivation of hop, a dual-purpose cannabaceae, is continuously expanding worldwide, where 62,111 ha were cultivated in 2020, with an increase of 769 ha over 2019 (+1.2%) [11]. Even Italy in recent years has registered a growing interest from farmers towards the cultivation of this plant, driven by the sudden development of the craft beer sector. In 2020, the Italian surface invested in hops was equal to a little more than 52 ha, with a greater concentration of production areas in the northern regions [11].

Hop cones contain several compounds that exert antimicrobial and antioxidant properties that have been exploited as preservative agents in the production of edible films [12,13] or to reduce spoilage during cold storage of meat [14]. To the best of our knowledge, no application of hop extracts to extend the shelf life of fresh-cut fruit has yet been made. Besides, there are several literature studies about the evaluation of edible and functionalized coatings of different fruit species, including kiwifruit [15]. Kiwifruit cultivation is one of the most important fruit and vegetable sectors in Italy, being the European leader and the third largest producer in the world [16]. Kiwifruit (*Actinidia deliciosa*) is highly appreciated by consumers due to its excellent nutraceutical profile, small caloric content, and high amount of vitamin C [17]. However, it is estimated that each year, due to size and appearance standards, about 15% of the crop is thrown away because the fruits are considered not suitable for direct consumption. In this context, the IV range processing of kiwifruit is an excellent solution to recover these by-products, which, due to inadequate shape or size, cannot be destined for the fresh fruit market. In this regard, Li et al. [18] demonstrated that the addition of polylysine (0.05%) to an alginate-based edible coating can preserve the pigments and morphological properties of fresh-cut kiwifruit, reducing microbial spoilage. Recently, sodium alginate (2% *m/m*) nanoemulsion coatings incorporated with ascorbic acid (0.5% *m/m*) and vanillin (from 0.5 to 1% *m/m*) showed the capacity to extend the shelf life of fresh-cut kiwifruit [19].

In light of these considerations, the aim of this study was to develop different ECs for ready-to-eat kiwifruit based on alginate functionalized with green extracts obtained from hop cones, investigating their effectiveness on the physico-chemical and sensory traits of kiwifruit slices, as well as their influence on the fruit nutritional quality by monitoring the changes in the total phenol, pigment, and ascorbic acid content during cold storage.

## 2. Materials and Methods

### 2.1. Chemicals

All used reagents were of analytical spectrophotometric grade. Folin–Ciocalteu’s reagent, sodium alginate (mannuronic to guluronic acid ratio: 1.33), calcium chloride, butylated hydroxytoluene (BHT), glycerol, calcium chloride, acetone, gallic acid, sodium carbonate, ascorbic acid, metaphosphoric acid, and EDTA were purchased from Sigma–Aldrich (Milan, Italy).

### 2.2. Plant Materials

Fresh fruits (*Actinidia deliciosa*, Hayward cv.; 10 kg) were provided by a local market and selected based on homogeneity in size, shape (gauge: 85–95), and apparent ripeness. The fruits were stored at 4 ± 1 °C and 70–75% relative humidity (RH) until used. Kiwifruit were sanitized with chlorinated water (200 mg L^−1^) for 5 min, rinsed with tap water and drained for 5 min, hand-peeled and then cut into 6–8 slices (group size) of 6 mm thickness with a stainless-steel slicer. The slices were randomly assigned to the different treatments to mitigate the effects of variations between fruits.

Wild seedless hop cones were obtained in dried form (containing about 8% by weight moisture) from random samples of the same accession present in the germoplasm collection field of the Lucanian Agency for the Development and Innovation in Agriculture (Potenza, Italy). Prior to be extracted, hop cones were grinded with a laboratory mill to a fine powder (sieve 0.5 mm) and kept under protection from light and humidity until analysis.

### 2.3. Preparation and Characterization of Plant Extracts

Dried hop cones were ultrasound (US) extracted with a hydroalcoholic food grade solvent mixture (H_2_O: ethanol: 50% *v*/*v*), according to Carbone et al. [10] without modifications. Hop bitter acids and xanthohumol were quantified (see Table 1) according to Carbone et al. [10], without modifications.

The ^1^H NMR profiling of HE used to functionalize ECs was carried out according to Carbone et al. [10] without modifications. Freeze-dried extracts were resuspended in 600 μL of deuterated solvent—methanol to water (1:1)—containing 0.5 mM of trimethylsilyl propionate-d4 (TSP) as the internal standard. After centrifugation, the supernatant was transferred into a 5 mm thin-walled glass NMR tube for subsequent spectral analysis. NMR spectra were collected by using a 700 MHz for ^1^H, equipped with a 5 mm inverse TXI probe and *z*-axis gradient. The ^1^H NMR spectra were acquired at 25 °C, with a spectral window of 15 ppm (carrier frequency at 4.7 ppm), 64 transients, and 2 steady-state scans, using 3 s of acquisition time and 2 s of relaxation delay. The ^1^H-^13^C HSQC (Heteronuclear Single-Quantum Correlation) experiment was acquired with a spectral window of 15 ppm × 100 ppm (carrier frequencies at 4.85 and 46.5 ppm) using 2048 × 600 data points and 36 transients for the aliphatic region. The collected spectra were processed and analyzed using Topspin software 2.0.

### 2.4. Preparation and Application of Functionalized ECs

In the present study, four different treatments were compared: (1) Control (CTRL) distilled water; (2) alginate 2% (*m*/*v*); (3) alginate 2% (*m*/*v*) + HE (0.5%; *v*/*v*); and (4) alginate 2% (*m*/*v*) + HE (1%; *v*/*v*). The concentrations of HE used to functionalize ECs have been chosen considering the potential contribution of hop bitterness to the coating, which was not revealed at these concentrations in preliminary studies conducted in our laboratory (data not shown). All coating solutions were prepared the same day as the sample dipping. The alginate coating solution was made by dissolving 2 g of sodium alginate in 100 mL of distilled water, with the addition of 1.5 g of glycerol as a plasticizer. The mixture was placed under mechanical stirring (750 rpm) at 70 °C for 1 h. The solution was then degassed by ultrasonic treatment (40 kHz) for 5 min and allowed to cool to room temperature before use.

To obtain a homogeneous coating of the samples, the kiwifruit slices were first immersed in their respective coating solutions (2 min), then allowed to dry on a steel grid for 1 min under a mild current of hot air, and then immersed again in a 2% (*m*/*v*) calcium chloride solution (2 min). The addition of divalent ions is mandatory to irreversibly form the alginate hydrogel needed to coat the fruit slices, inducing the gelling mechanism and crosslinking reaction. The samples were then left to dry completely before packaging.

Packages were stored at 4 ± 0.5 °C and 90% RH for 10d. All analyzed parameters were evaluated at the beginning of the experiment (after coating/dipping: t0) and at 2, 5, 7, and 10 d after cool storage, on 6 slices per replicate for treatment (4 treatments × 5 time of storage × 5 replicates: 100 boxes).

### 2.5. Quality Traits of Fresh-Cut Kiwifruit

Weight loss (WL) was calculated by weighting individual boxes immediately after the treatment (t0) and at different sampling times. The data were expressed in terms of percentage of weight reduction with respect to the initial time (t0). All of the following analyses were performed on kiwifruit slices homogenized with an Ultra-Turrax blender (Ultra Turrax T25, IKA, Milan, Italy) at 9000 rpm as for nutraceutical analysis. Total soluble solid (TSS) content was determined using a digital refractometer (Refracto 30 PX, Mettler Toledo, Milan, Italy) on homogenized samples centrifuged at 5000× *g* for 10 min and filtered through a Whatman no. 41 filter paper; data are given as °Brix. Titratable acidity (TA) was measured by potentiometric titration against 0.1 N NaOH to a pH 8.1 endpoint and expressed as percent citric acid [20].

The pH value was measured using a digital pH-meter (785 DMP, Methrom, Milan, Italy). The kiwifruit slice color was assessed using a Minolta colorimeter (CR5, Minolta Camera Co., Osaka, Japan) equipped with a D65 illuminant, in the CIELAB space. The chromaticity values (L: lightness; a*: green to red; b*: blue to yellow) were evaluated on the opposite sides of three fruit slices from each tray per treatment.

### 2.6. Nutraceutical Traits of Fresh-Cut Kiwifruit

All of the following analyses were performed on fruit homogenized with an Ultra-Turrax blender (Ultra Turrax T25, IKA, Milan, Italy) at 9000 rpm, in an ice bath and in the dark to avoid the degradation of sample phytochemicals. Ascorbic acid content (AAC) was determined according to Ciccoritti et al. [21], with some modifications. Briefly, 5 g of homogenate was extracted with 20 mL of a solution of metaphosphoric acid (HPO_3_; 16% *m*/*v*) and 0.18% (*m*/*v*) disodium ethylenediamine tetra acetic acid (EDTA). The suspension was then placed under agitation for 10 min at room temperature and in the dark, centrifuged at 5000× *g* for 10 min at 4 °C, filtered, and the supernatant was collected to be further analyzed. Ascorbic acid (AA) was measured at 760 nm using a UV–vis spectrophotometer (model 6300 PC, VWR, Milan, Italy). AAC was calculated using an AA calibration curve, and the results were expressed in milligrams of ascorbic acid per 100 g of fresh matter.

Total polyphenol content (TPC) was determined according to Carbone et al. [22] with some modifications. Briefly, to 1 g of sample homogenate, 5 mL of extracting solution (MeOH: H_2_O = 80: 20 (*v*/*v*); containing 0.1% (*m*/*v*) HCl) was added. The resulting suspension was subjected to mechanical agitation (300 rpm) for 30 min, at room temperature and protected from light, then it was sonicated for 30 min and centrifuged at 5000× *g* for 15 min. At this point, the supernatant (extract) was recovered and stored at −20 °C until analysis. TPC was calculated using a gallic acid calibration curve. Results were expressed as milligram of gallic acid equivalents (GAE) per 100 g of fresh weight (mg GAE 100 g^1^ fw).

The content of total carotenoids (TC) and chlorophylls (chlorophyll a, chla; chlorophyll b, chlb; and total chlorophyll, chl) was determined spectrophotometrically after extracting fruit homogenates according to Benlloch-Tinoco et al. [23], with some modifications. Briefly, 10 mL of cold acetone solution (4 °C) containing 0.01% (*m*/*v*) butylhydroxytoluene (BHT) was added to 1 g of homogenate. The suspension was subjected to mechanical agitation (300 rpm) for 15 min, at room temperature and protected from light. The extract was filtered through Whatman no. 1 paper immediately prior to the analyses. Pigments were determined spectrophotometrically on a UV-vis spectrophotometer (model 6300 PC, VWR, Milan, Italy) set at different wavelengths simultaneously (λ: 663, 648 and 470 nm for chla, chlb, total carotenoids, respectively), and their content was calculated according to Lichtenthaler and Buschmann [24]. Results were expressed in μg 100 g^−1^ fw.

### 2.7. Visual Appearance of Fresh-Cut Kiwifruit

The visual appearance of the samples that were analyzed were evaluated according to the method described by Chiabrando and Giacalone [25], without modifications. Briefly, a lab panel (eight panelists: five women and three men; age: 27–55) trained on kiwifruit sensorial analysis scored samples, using a scale in which 9 = excellent quality; 7 = good quality; 5 = fair quality (limit of marketability); 3 = poor quality (limit of edibility); and 1 = very bad quality.

### 2.8. Statistical Analysis

Described analyses were carried out in triplicate, and the statistical analysis of the data, expressed as mean ± standard deviation (sd), was performed with the SPSS 24.0 software (SPSS, Inc., Chicago, IL, USA). A preliminary exploratory analysis of the experimental data was performed to check for normality of distribution (Kolmogorov-Smirnov test) and equality of variances (Levene test). In order to reduce the impact of any outliers detected in the data, we replaced them with the mean plus two standard deviations [26]. Analysis of variance (ANOVA) was then performed on the analytical data using a completely randomized factorial experimental design, with two factors (treatment (T) and storage time (t)). Mean comparisons were performed using Duncan’s multiple range test (*p* < 0.05).

## 3. Results

### 3.1. Chemical Composition of Hop Extract

The chemical composition of HE used to functionalize ECs is presented in Table 1. The extract was characterized by its TPC and nutraceutical profile. Several bioactive compounds were present in the extract—mainly alpha and beta acids, known for their antimicrobial and antifungal power—and also xanthohumol, a prenylated flavonoid with well-known beneficial effects for human health [27], which was also proven to inhibit microbial growth [14].

In this study, a combination of ^1^H-NMR and ^1^H-^13^C HSQC experiments was also applied to investigate the overall phytomic profiling of HE used in coating formulations. The ^1^H-^13^C HSQC experiment provides correlations between a carbon and its attached protons, allowing a 2D heteronuclear chemical shift correlation map to be obtained between directly bonded ^1^H and X-heteronuclei (i.e., ^13^C).

**Figure 1:**
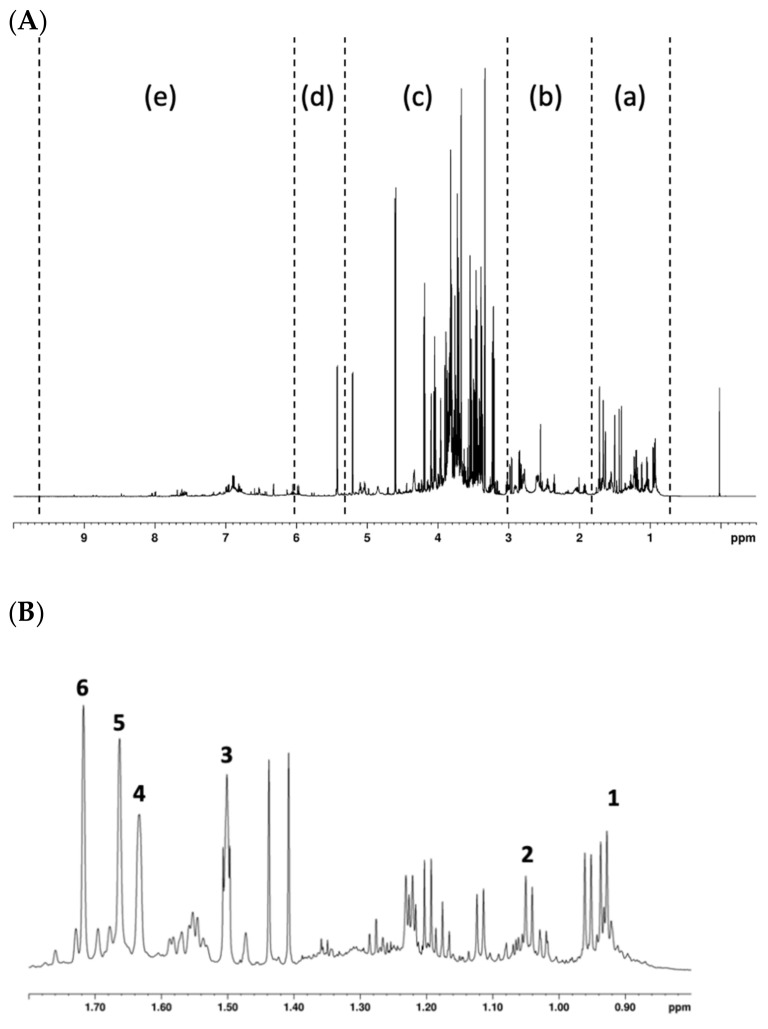
The ^1^H NMR spectrum of hop extract. Section (**A**): (a) hop bitter acids region from 0.5 to 1.8 ppm; (b) aliphatic region from 1.8 to 3 ppm; (c) sugars from 3.0 to 5.5 ppm; (d) terpenes from 5.5 to 6.0 ppm; and (e) aromatics from 6.0 to 9.5 ppm. Section (**B**): the enlargement of the bitter acids region is shown in this section with the assigned signals of humulone (1, 3–6) and cohumulone (2).

The ^1^H NMR spectrum of the hop extract resulted rich in signals that contribute to defining a complex fingerprint (Figure 1A). The spectrum was divided into five regions: aromatic, terpenes, sugars, aliphatic, and hop bitter acids. As for the latter, signals related to two alpha acids, cohumulone and humulone, were distinguished (Figure 1B). The assignment was confirmed by acquiring the ^1^H-^13^C HSQC experiment in which the chemical shifts of -CH_2_ and -CH_3_ groups of these two α-acids were measured.

**Figure 2:**
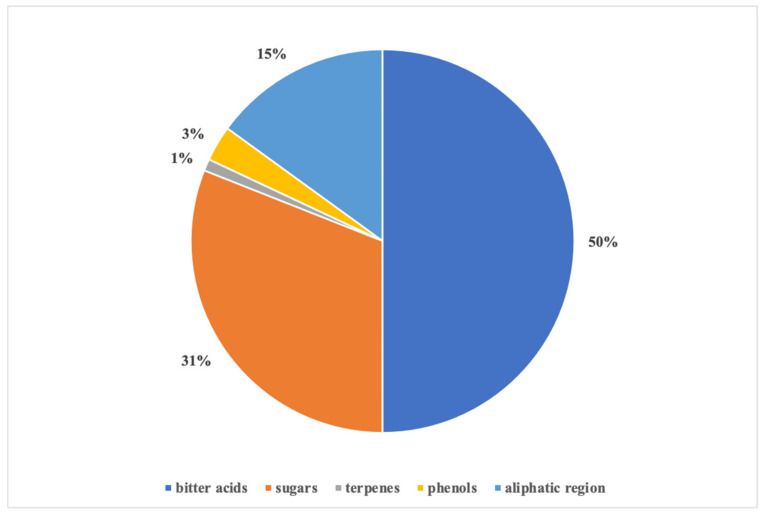
Relative composition (area %; *n* = 3) of hop extract by ^1^HNMR analysis.

The contribution of each region, expressed as area percentage of the integrated NMR signals, is shown in Figure 2. The bitter acid zone contributed 50% compared to all the other zones of the spectrum, thus demonstrating the abundance of these compounds in the extract. In particular, the ratio between cohumulone and humulone integrals indicated that the latter was present in the extract twice as much as the other α-acid.

### 3.2. Influence of Active ECs on the Fresh-Cut Kiwifruit Quality Parameters

**Figure 3:**
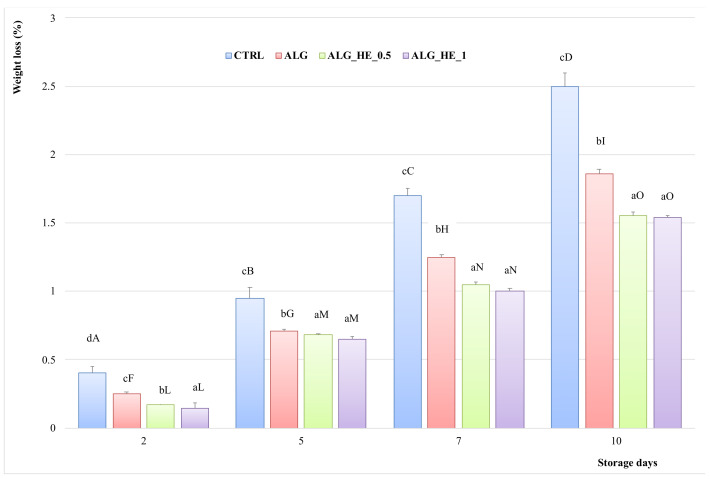
Percentage of weight loss (mean ± sd; *n* = 5) of fresh-cut kiwifruit underwent different treatments during storage at 4 °C. CTRL, control samples; ALG, alginate samples; ALG_HE_0.5, samples coated with alginate added with 0.5% of hop extract; ALG_HE_1, samples coated with alginate added with 1% of hop extract. Different lower-case letters indicate significant differences (*p* < 0.05) among treatments at the same storage time. Different upper-case letters indicate significant differences (*p* < 0.05) in each treatment over the storage time.

Figure 3 shows the weight loss (%) of fresh-cut kiwifruit subjected to different treatments during cold storage. For both untreated and treated samples, weight loss (%) increased almost linearly during storage time until reaching a maximum value at the end of the experimental trial. However, when evaluating the behavior of different ECs compared to the control, it can be observed that all treatments had a positive effect (*p* < 0.05) on weight loss, thus preserving water loss in the product. At the end of the trials (10d), CTRL samples showed the highest weight loss (2.5%; *p* < 0.05). In contrast, functionalized ECs showed the lowest mass loss (1.55 and 1.54% for ALG_HE_0.5 and ALG_HE_1, respectively; *p* < 0.05), indicating that not only the hydrocolloid but also the bioactive constituents play a role in preserving the samples from mass loss, in accordance with previous studies [28].

**Figure 4:**
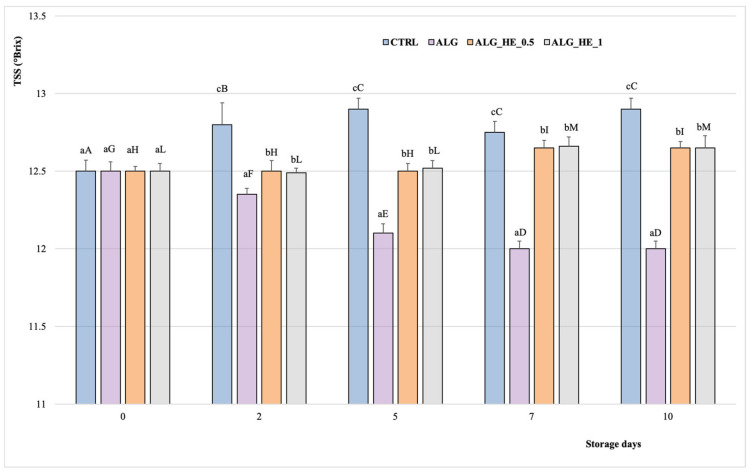
Changes in total soluble solids (°Brix; mean ± sd; n = 5) of fresh-cut kiwifruit underwent different treatments during storage at 4 °C. TSS, total soluble solids; CTRL, control samples; ALG, alginate samples; ALG_HE_0.5, samples coated with alginate added with 0.5% of hop extract; ALG_HE_1, samples coated with alginate added with 1% of hop extract. Different lower-case letters indicate significant differences (*p* < 0.05) among treatments at the same storage time. Different upper-case letters indicate significant differences (*p* < 0.05) in each treatment over the storage time.

TSS and TA are among the main parameters in the assessment of fruit quality, as they influenced the taste and sensory profile of the fruit [29]. According to literature studies, TSS in fruits increases during storage while TA decreases [30]. Figure 4 shows the TSS content of samples analyzed during cold storage. The overall TSS of the analyzed samples increased gradually during storage to a different extent depending on the treatment applied (*p* < 0.05). The CTRL samples showed the highest TSS content (12.9 °Brix), reached after five days of cold storage, which remained constant until the end of the test. In contrast, no significant differences were found in kiwifruit slices coated with different HE-functionalized alginates up to the fifth day of storage (12.5 °Brix), when there was a slight but significant increase in TSS compared to previous values (*p* < 0.05), with no significant differences between the different concentrations of plant extract used (TSS at the end of storage: 12.65 °Brix for both ALG_HE_0.5 and ALG_HE_1 samples), according to literature data [19]. These features highlight the role of EC in reducing the rates of carbohydrate breakdown, delaying fruit ripening.

As far as TA is concerned, no significant differences were observed between ALG_HE samples over the length of the cold storage, regardless of the concentration of the plant extract used (Table 2). Besides, CTRL samples showed a decreasing trend in TA values, reaching the lowest value recorded during the test on the tenth day of cold storage (1.38 ± 0.03% citric acid; *p* < 0.05). The pH significantly increased during storage for both untreated and alginate-coated samples (*p* < 0.05), although in the latter case there was a smaller increase. In contrast, no significant differences were found in the pH of samples coated with HE-functionalized alginate throughout the cold storage period, regardless of the concentration of plant extract used (Table 2). These features highlight the effectiveness of EC in delaying fruit ripening by reducing acidity loss as a result of reduced respiratory metabolism during storage [30,31].

Fruit color is an important index to evaluate the quality and ripening process during postharvest, but it is also an important extrinsic indicator in the consumers’ perception of quality, making minimal color change desirable when applying a coating. In the present study, changes in flesh color of samples were monitored by measuring CIELAB coordinates L, a*, and b*.

**Figure 5:**
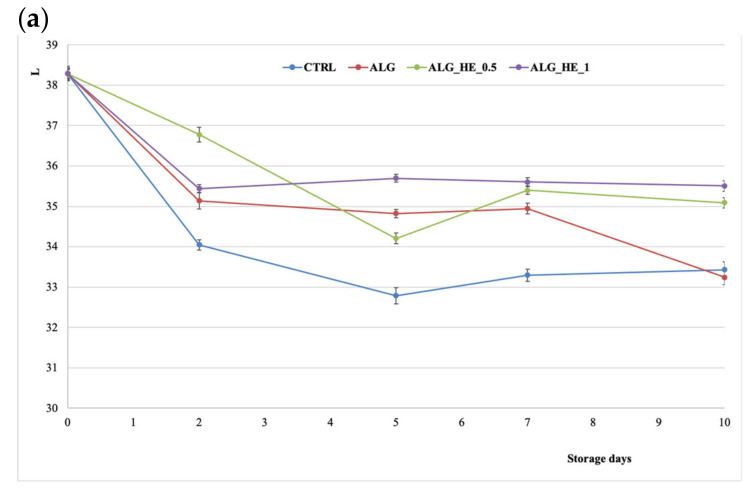

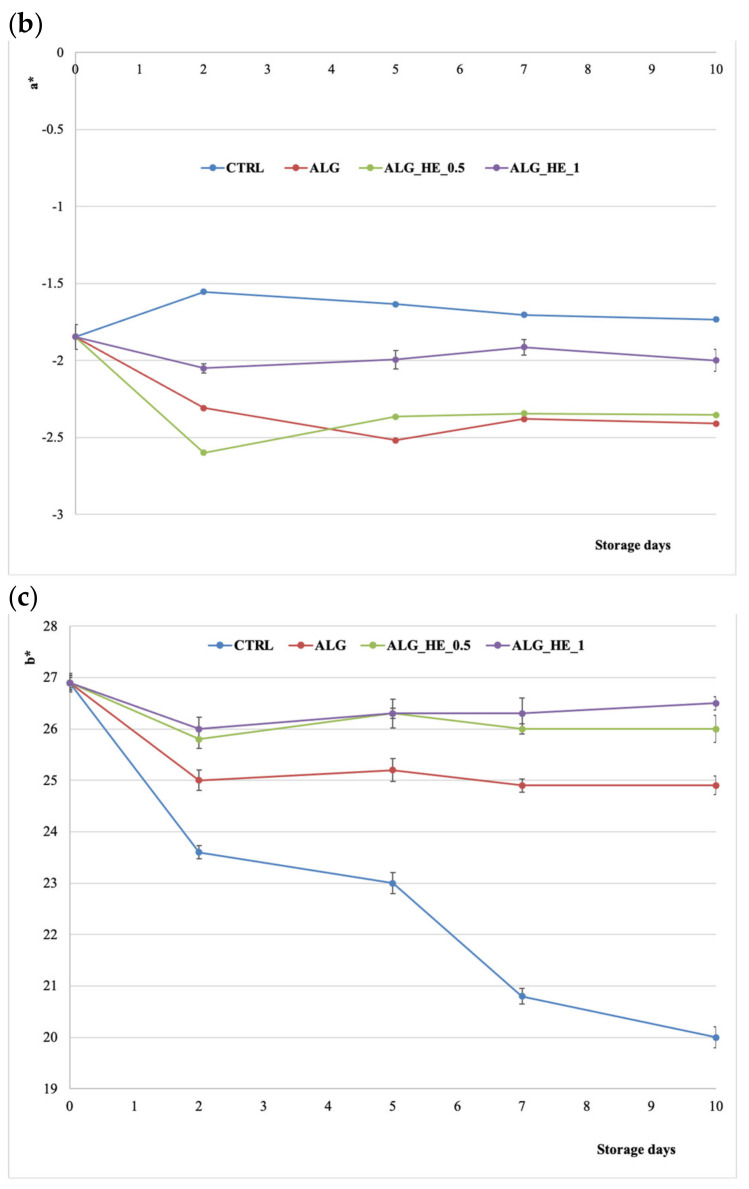
Changes in CIELAB coordinates (mean ± sd; *n = 30*) of fresh-cut kiwifruit underwent different treatments during storage at 4 °C: (**a**) effect on L coordinate; (**b**) effect on a* coordinate; (**c**) effect on b* coordinate. CTRL, control samples; ALG, alginate samples; ALG_HE_0.5, samples coated with alginate added with 0.5% of hop extract; ALG_HE_1, samples coated with alginate added with 1% of hop extract.

The lightness in both treated and untreated fruits decreased over the storage period significantly (*p* < 0.05), by a different extent depending on the treatment applied (Figure 5a). From the inferential analysis of the data, the CIELAB L* coordinate was found to be statistically influenced by both independent variables (T and t) and their interaction (T × t), highlighting, with regard to the treatments performed, the presence of two homogeneous subsets: CTRL and ALG vs. ALG_HEs, which were both able to preserve the samples from browning better than alginate alone (*p* < 0.05).

Kiwifruit is characterized by a brilliant green color, due to the presence of chlorophyll, which tends to be lost with oxidation. Figure 5b shows the trends of coordinate a* in the CIELAB space. The length of cold storage statistically influenced (*p* < 0.05) the value of a* during the first five days, regardless of the type of treatment applied. Untreated samples showed a significant increase in the a* coordinate during the experimental trial (*p* < 0.05), confirming the triggering of browning and oxidation phenomena of the kiwifruit slices following cold storage. As for the coatings, all of the treatments tested were proven to be able to counteract the increase of the a* coordinate. However, ALG and ALG_HE_0.5 had a significant (*p* < 0.05) greener color and lower a* values compared to the other samples, highlighting the ability of these coatings to preserve samples from oxidative degradation. Interestingly, increasing the concentration of the plant extract did not produce a positive effect on the a* value, which was statistically similar to that of the control at the end of storage. A similar inverse dose response was also reported by Li et al. [18], who treated fresh-cut kiwifruit with an alginate coating functionalized with increasing concentrations of poly-ε-lysine.

Figure 5c shows the effect of ECs on coordinate b* of the kiwifruit samples during cold storage. This parameter, in the case of the CTRL samples, tended to decrease almost linearly over the cold storage, while samples coated with functionalized alginate did not show statistically significant variations for this parameter between the beginning and the end of the trial, regardless of the concentration of HE used. These features are in line with those reported by Hassani, Garousi, and Javanmard [32].

The most performing effect shown by the coatings enriched with hop plant extract on fruit color preservation suggests that the antioxidant potential and antimicrobial activity of the extract, due to the presence of hop bitter acids and polyphenols, may have acted more effectively in inhibiting metabolic activity such as enzymatic browning responsible for the color changes in pulp fruits, as compared to CTRL and ALG treatments.

### 3.3. Influence of Active ECs on the Fresh-Cut Kiwifruit Nutraceutical Parameters

**Figure 6:**
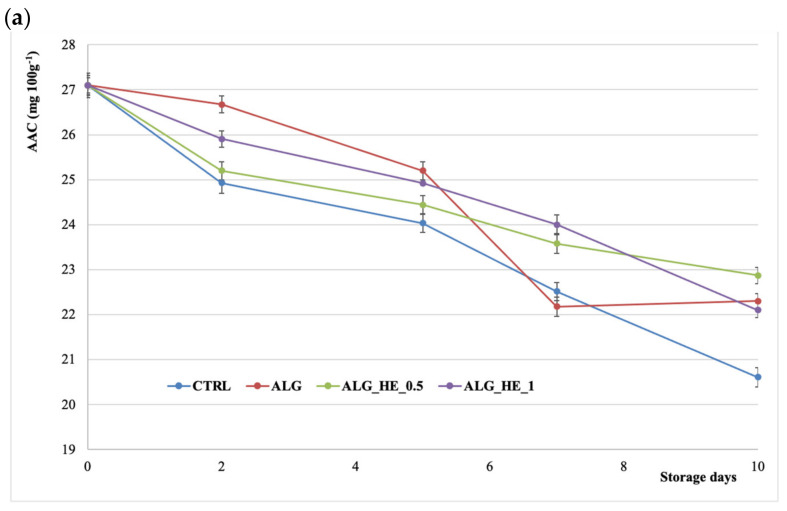

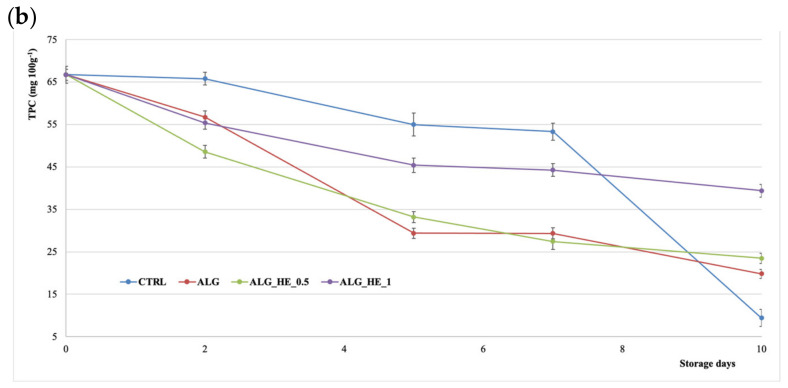
Changes in (**a**) ascorbic acid content (AAC) and (**b**) total polyphenol content (TPC) of fresh-cut kiwifruit underwent different treatments during storage at 4 °C (mean ± sd; *n* = 5). CTRL, control samples; ALG, alginate samples; ALG_HE_0.5, samples coated with alginate added with 0.5% of hop extract; ALG_HE_1, samples coated with alginate added with 1% of hop extract.

The high nutritional value of kiwifruit is due to its high vitamin C (ascorbic acid) content, which can be altered by postharvest handling procedures. Figure 6a shows the effect of treatments applied on AAC of fresh-cut kiwifruit. AAC decreased over time for all samples analyzed and both coating and storage time statistically influenced its content (*p* < 0.05), as also reported by Chiabrando and Giacalone [25] for fresh-cut Jintao kiwifruit. CTRL samples showed the highest loss of AA at the end of cold storage (−24%), while samples coated with ALG_HE_0.5 preserved more than 84% of their initial AAC. In addition, no statistical differences for AAC were observed among all coated samples at the end of the storage, highlighting the effectiveness of the applied coatings in lowering the package headspace percent of O_2_ and, as a consequence, in deleting the oxidative degradation of vitamin C in kiwifruit slices [33].

Figure 6b shows the TPC of samples analyzed during storage. During the shelf-life period, a decrease in TPC values—influenced both by coatings and storage time—was observed (*p* < 0.05), as also reported in the literature [31]. CTRL samples showed a contained loss of TPC in the first week to collapse at the end of the storage (−86%). All tested ECs showed a lower TPC reduction as compared to the CTRL, with the highest final TPC recorded for the ALG_HE_1 sample (39.9 mg GAE 100 g^−1^ fw; −41% of initial TPC).

**Figure 7:**
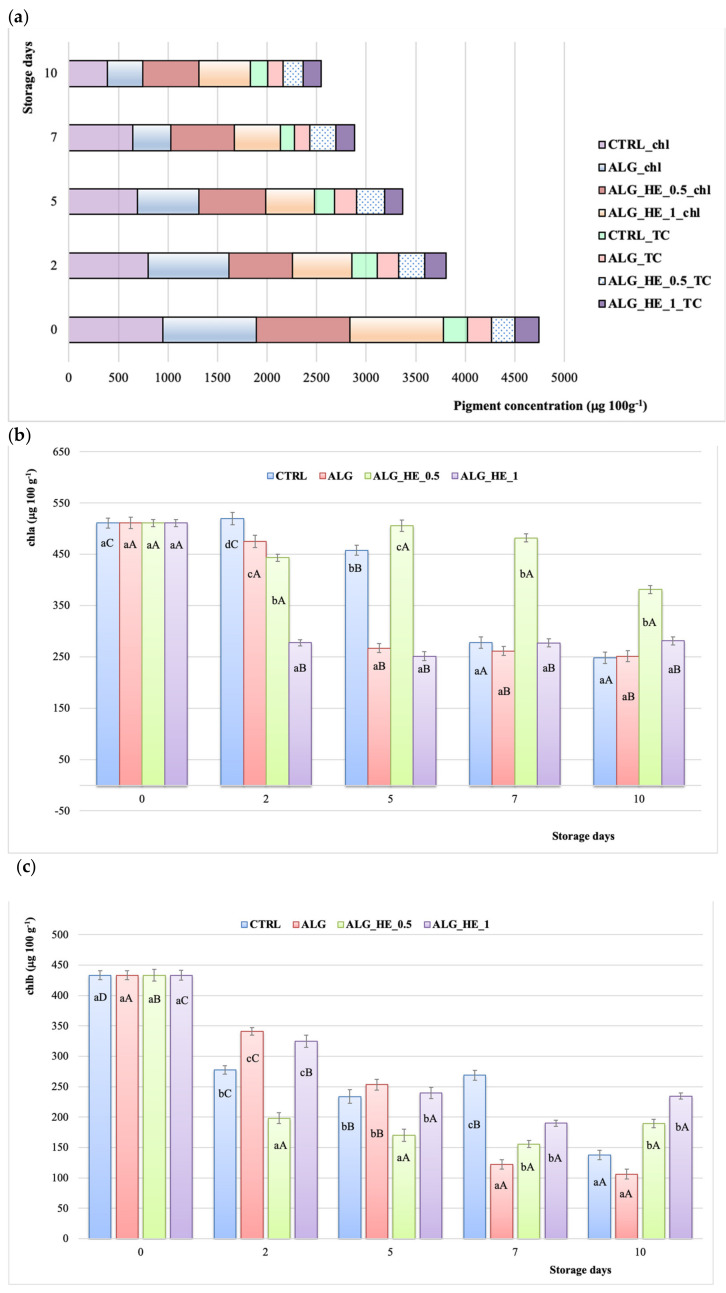
Changes in pigment content of fresh-cut kiwifruit underwent different treatments during storage at 4 °C (mean ± sd; *n* = 5). (**a**) Total chlorophylls (chl) and total carotenoids (TC); (**b**) chlorophyll a content (chla); (**c**) chlorophyll b content (chlb). CTRL, control samples; ALG, alginate samples; ALG_HE_0.5, samples coated with alginate added with 0.5% of hop extract; ALG_HE_1, samples coated with alginate added with 1% of hop extract. Different lower-case letters indicate significant differences (*p* < 0.05) among treatments at the same storage time. Different upper-case letters indicate significant differences (*p* < 0.05) in each treatment over the storage time.

Chlorophylls in kiwifruit (*A. deliciosa*) are one of the main pigments that contribute to the characteristic bright green color of its flesh, which is crucial for the consumers’ sensory acceptance, being their main determinant of quality. Moreover, pigments are reported to exert a plethora of health beneficial effects [34]. In the present study, chl of samples analyzed decreased almost linearly for both CTRL and ALG ones, with a reduction of 59.2% and 62.2%, respectively, at the end of the storage (*p* < 0.05; Figure 7a). Besides, both functionalized alginate coatings were able to preserve better chl content, with a final reduction of its content of 39.6% and 45.4% for ALG_HE_0.5 and ALG_HE_1, respectively (Figure 7a). These results are in line with those reported by Fan et al. [28], who observed greater chlorophyll retention at the end of the storage period for samples coated with plant extract-functionalized hydrocolloid as compared to others. As far as chla is concerned (Figure 7b), its content decreased significantly in all samples starting from the fifth day of cold storage, except for the ALG_HE_0.5 ones for which a significant effect of pigment containment was observed up until the end of the trial (+74.6%, *p* < 0.05). It is interesting to note that the increasing of the HE concentration produced a significant loss of this pigment in the first two days of storage (−45.70%; *p* < 0.05), and then remained constant over time, with final values statistically similar to those of the other samples tested (280.94 μg 100g^−1^ fw). As far as chlb is concerned (Figure 7c), functionalized alginate coatings showed a better preserving action with a chlb retention at the end of the storage of 43.75% and 54.07% for ALG_HE_0.5 and ALG_HE_1, respectively.

Among the complex network of minor compounds that may also be associated with beneficial physiological functions of kiwifruit are carotenoids (i.e., lutein, zeaxanthin, and β-carotene), which can also be considered as an index of ripening during storage [35]. In contrast to what was observed for total chlorophyll, treatments did not statistically influence the TCC of samples analyzed (Figure 7a), apart from those coated with ALG_HE_0.5 that showed the highest retention of carotenoids at the end of the storage (+83.5%; *p* < 0.05). To the best of our knowledge, there is no literature data about the influence of ECs on TCC of fresh-cut kiwifruit.

### 3.4. Influence of Active ECs on the Fresh-Cut Kiwifruit Visual Appearance

**Figure 8:**
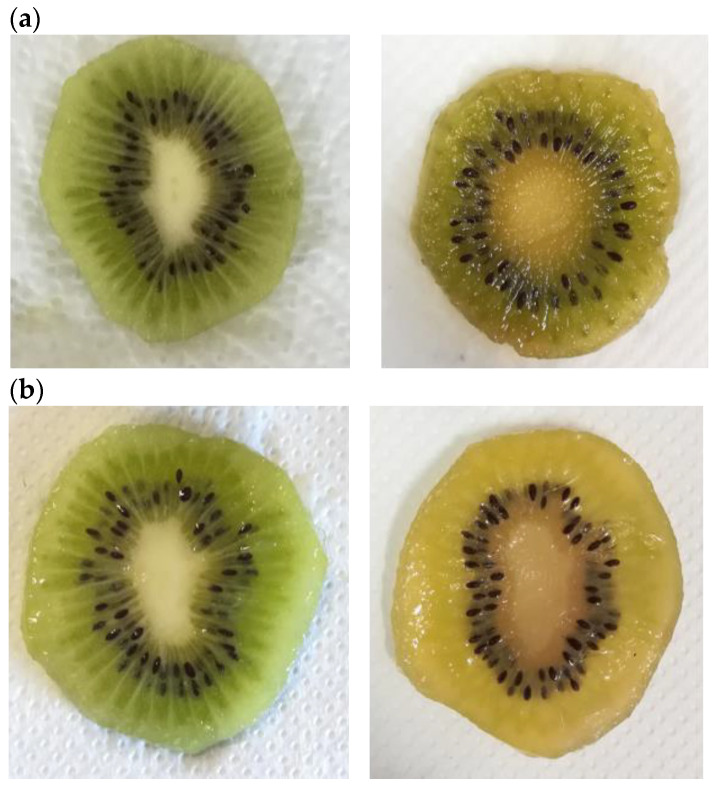

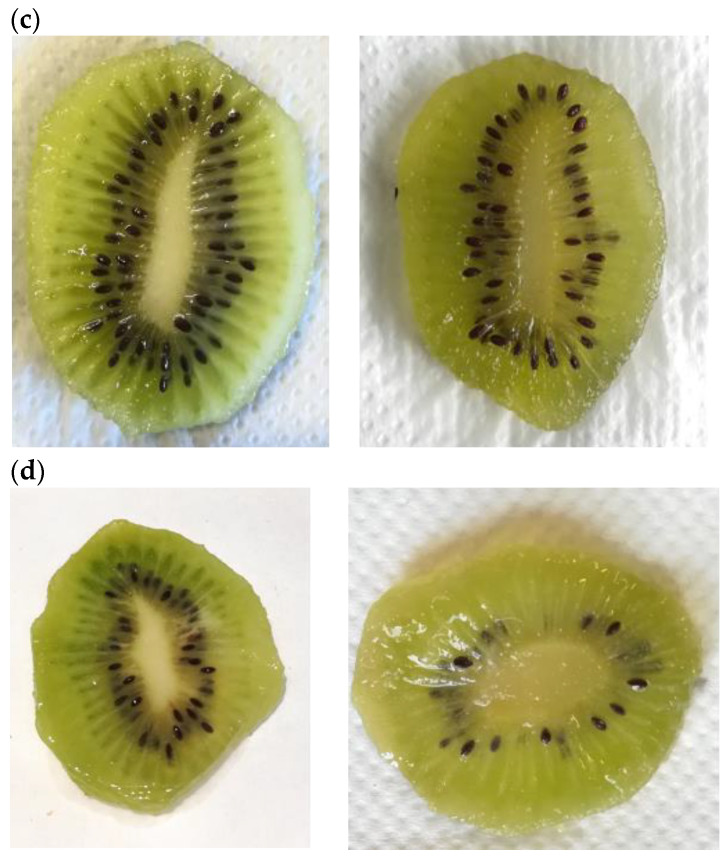
Visual appearance of fresh-cut kiwifruit at the beginning (on the left side) and at the end (on the right side) of the storage. (**a**) CTRL, control samples; (**b**) ALG, alginate samples; (**c**) ALG_HE_0.5, samples coated with alginate added with 0.5% of hop extract; and (**d**) ALG_HE_1, samples coated with alginate added with 1% of hop extract.

Fresh-cut fruit is very perishable, and the main factors affecting the loss of consumer’s acceptability are discoloration or browning, dryness, and loss of texture. These parameters determine visual appearance, which influences the consumer’s perception and thus the level of acceptability to purchase, as consumers associate desirable internal quality characteristics with external appearance [36]. Figure 8a–d shows the visual appearance of samples at the beginning of the trial (left) and after 10 days of cold storage (right), highlighting the positive effect of functionalized coating on the visual traits of kiwifruit slices (ALG_HE_0.5 and ALG_HE_1, in pictures c and d, respectively). These findings are in line with literature data, reporting a preservative effect of an alginate functionalized coating with antioxidants and antimicrobials in maintaining the size and light green color of kiwifruit slices during cold storage [19].

Table 3 shows the scores assigned by the lab panelists in the visual test [25]. The highest visual scores, underlining the good quality of slices at the end of the cold storage, were recorded for both alginate functionalized coatings, with non-statistical differences between samples. Untreated samples were considered by the panel to be of poor quality, mainly based on the dry appearance of the slices at the end of the trial. Unfortunately, the impossibility of being able to analyze the microbial load and the types of microorganisms that could potentially be present in the analyzed samples, even though they are not detectable to the visual analysis, did not allow us to perform the tasting tests, which will hopefully be set up in future studies.

## 4. Conclusions

Color changes (i.e., enzymatic browning) and weight loss are the two main processes that contribute to quality changes in fresh-cut products, perceived by consumers as freshness indicators. In this study, the application of edible alginate coatings with the addition of HE at different concentrations was evaluated as a tool to delay quality and nutraceutical changes in fresh-cut kiwifruit. The results revealed the positive influence of adding hop cone extract to the alginate coating of freshly-cut kiwifruit on their physicochemical and nutraceutical traits during cold storage. The effect of HE was found mainly in its ability to retain the main classes of biologically active compounds (i.e., polyphenols, pigments), and especially in the retention of vitamin C compared to the control (preservation of more than 84% of kiwifruit initial AAC), the latter being one of the nutritional aspects of major importance in favor of a wider consumption of fresh kiwifruit. Among the hop concentrations tested, coatings containing 0.5% (*v*/*v*) HE demonstrated better pigments and vitamin C retention, nominating themselves as the best coatings among those tested. The presence of several bioactive compounds in HEs with antioxidant and antimicrobial activities were able to preserve the freshness of the fruits, providing a valuable tool to prolong their shelf-life with respect to aspects related to the main quality attributes, nutraceutical parameters, and product appearance. However, the influence of coatings and plant extracts on the development of microbial load and on sensorial aspects related to taste and aroma still needs to be investigated in more detail.

Nevertheless, the addition of HE rich in bioactive compounds—mainly hop bitter acids and xanthohumol—to the edible-coatings has proven to be a promising approach for enhancing the quality and safety of fresh-cut products. Further studies should also consider the exploitation of hop by-product to obtain these extracts, thus applying a circular economy approach. The industrial exploitation of the results obtained, which can also be validated on other fruits, could stimulate the consumption of fresh fruit with an optimal nutritional and antioxidant profile that would otherwise be lost, also in view of the fact that the sliced kiwifruit is one of the most used fruits in confectionery and food preparations (e.g., ready-made fruit salads). In addition, the production of ready-to-eat kiwifruit, capable of preserving high nutritional and nutraceutical characteristics over time, would be a valid alternative to exploit the benefits of less valuable fruits in terms of size and shape, which are currently considered wasteful and are poorly paid for, thus generating a positive economic impact on the entire production chain.

## Figures and Tables

**Table 1:** Chemical composition of hop extract (mean ± sd; *n* = 3).

Parameters	Values
α acids (%)	3.3 ± 0.1
β acids (%)	3.7 ± 0.1
Xanthohumol (mg g^−1^)	4.3 ± 0.1
Total phenolic content (mg GAE g^−1^)	12.9 ± 0.9

^1^H NMR Phytomic Profiling of HE.

**Table 2:** Effects of analyzed edible coatings on pH and titratable acidity of fresh-cut kiwifruit (mean ± sd; *n* = 5).

Treatment	Storage Days									
	pH					TA ^1^				
	0	2	5	7	10	0	2	5	7	10
CTRL ^2^	3.17 ± 0.01 aA	3.18 ± 0.04 aB	3.28 ± 0.01 bC	3.35 ± 0.01 cC	3.35 ± 0.03 cC	1.43 ± 0.05 bA	1.39 ± 0.02 aA	1.39 ± 0.04 aA	1.41 ± 0.05 aB	1.38 ± 0.03 aA
ALG ^3^	3.17 ± 0.01 aA	3.21 ± 0.01 bC	3.25 ± 0.01 bB	3.24 ± 0.01 bB	3.24 ± 0.01 bB	1.43 ± 0.07 aA	1.43 ± 0.05 aB	1.45 ± 0.06 aB	1.45 ± 0.02 aA	1.47 ± 0.05 aB
ALG_HE_0.5 ^4^	3.17 ± 0.02 aA	3.15 ± 0.01 aA	3.16 ± 0.04 aA	3.18 ± 0.01 aA	3.17 ± 0.01 aA	1.43 ± 0.05 aA	1.41 ± 0.05 aB	1.41 ± 0.05 aB	1.45 ± 0.01 aA	1.43 ± 0.05 aB
ALG_HE_1 ^5^	3.17 ± 0.01 aA	3.17 ± 0.01 aA	3.18 ± 0.02 aA	3.17 ± 0.01 aA	3.19 ± 0.02 aA	1.43 ± 0.04 aA	1.43 ± 0.02 aB	1.44 ± 0.02 aB	1.40 ± 0.04 aA	1.43 ± 0.05 aB

^1^ TA: titratable acidity, data are expressed in terms of % citric acid. ^2^ CTRL: control, uncoated samples. ^3^ ALG: samples coated with alginate 2% (*m*/*v*). ^4^ ALG_HE: samples coated with alginate 2% (*m*/*v*) functionalized with hop extract (0.5% *v*/*v*). ^5^ ALG_HE_1: samples coated with alginate 2% (*m*/*v*) functionalized with hop extract (1% *v*/*v*). In each row, different lower-case letters indicate significant differences (*p* < 0.05). In each column, different upper-case letters indicate significant differences (*p* < 0.05).

**Table 3:** Appearance scores of samples analyzed (mean ± sd; *n* = 30).

	Storage Time (d)	
Sample	0	10
CTRL	7.7 ± 0.3 bA	3.1 ± 0.1 aA
ALG	8.6 ± 0.1 bB	6.5 ± 0.1 aB
ALG_HE_0.5	8.9 ± 0.1 bB	7.1 ± 0.2 aC
ALG_HE_1	8.6 ± 0.3 aB	7.3 ± 0.1 aC

In each row, different lower-case letters indicate significant differences (*p* < 0.05). In each column, different upper-case letters indicate significant differences (*p* < 0.05).

## Data Availability

All data is contained within the article.
